# Supplementary material for: Comparative multiomics analysis of cell physiological state after culture in a basket bioreactor
Source: Sci Rep. 2022 Nov 23;12:20161. doi: 10.1038/s41598-022-24687-4 (PMC9686226; doi:10.1038/s41598-022-24687-4)
Supplement: Supplementary file 1 — Supplementary Information 1. [file 41598_2022_24687_MOESM1_ESM.zip › raw data/Transcriptomic raw data/6.Enrichment/2.KEGG/TE1vsTC1/GSEA/CYTOKINE_CYTOKINE_RECEPTOR_INTERACTION(CSAB04060).html]

Details for gene set CYTOKINE\_CYTOKINE\_RECEPTOR\_INTERACTION(CSAB04060)[GSEA]

|  || Dataset | readcount.sample |
| Phenotype | sample.cls |
| Upregulated in class | TE1 |
| GeneSet | CYTOKINE\_CYTOKINE\_RECEPTOR\_INTERACTION(CSAB04060) |
| Enrichment Score (ES) | 0.66418177 |
| Normalized Enrichment Score (NES) | 0.98802114 |
| Nominal p-value | 0.33333334 |
| FDR q-value | 1.0 |
| FWER p-Value | 1.0 |
Table: GSEA Results Summary

  

Fig 1: Enrichment plot: CYTOKINE\_CYTOKINE\_RECEPTOR\_INTERACTION(CSAB04060)      
 Profile of the Running ES Score & Positions of GeneSet Members on the Rank Ordered List

  

| PROBE | DESCRIPTION (from dataset) | GENE SYMBOL | GENE\_TITLE | RANK IN GENE LIST | RANK METRIC SCORE | RUNNING ES | CORE ENRICHMENT || 1 | novel.64 | - |  |  | 7523 | 1.179 | 0.6642 | Yes |
Table: GSEA details [plain text format]

  

Fig 2: CYTOKINE\_CYTOKINE\_RECEPTOR\_INTERACTION(CSAB04060)      
 Blue-Pink O' Gram in the Space of the Analyzed GeneSet

  

Fig 3: CYTOKINE\_CYTOKINE\_RECEPTOR\_INTERACTION(CSAB04060): Random ES distribution      
 Gene set null distribution of ES for **CYTOKINE\_CYTOKINE\_RECEPTOR\_INTERACTION(CSAB04060)**
